# EZH2 Promotes Malignant Behaviors via Cell Cycle Dysregulation and Its mRNA Level Associates with Prognosis of Patient with Non-Small Cell Lung Cancer

**DOI:** 10.1371/journal.pone.0052984

**Published:** 2012-12-31

**Authors:** Wei Cao, Rachel de Oliveira Ribeiro, Diane Liu, Pierre Saintigny, Ronghui Xia, Yuwen Xue, Ruxian Lin, Li Mao, Hening Ren

**Affiliations:** 1 Shanghai Key Laboratory of Stomatology, Department of Oral Maxillofacial-Head and Neck Oncology, Ninth People’s Hospital, Shanghai Jiao Tong University School of Medicine, Shanghai, China; 2 Department of Oncology and Diagnostic Sciences, University of Maryland Dental School, Baltimore, Maryland, United States of America; 3 Department of Thoracic/Head and Neck Medical Oncology, The University of Texas MD Anderson Cancer Center, Houston, Texas, United States of America; 4 Bioinformatics and Computational Biology, The University of Texas MD Anderson Cancer Center, Houston, Texas, United States of America; The University of Texas MD Anderson Cancer Center, United States of America

## Abstract

**Background:**

Epigenetic silencing is a common mechanism to inactivate tumor suppressor genes during carcinogenesis. Enhancer of Zeste 2 (EZH2) is the histone methyltransferase subunit in polycomb repressive complex 2 which mediates transcriptional repression through histone methylation. EZH2 overexpression has been linked to aggressive phenotypes of certain cancers. However, the mechanism that EZH2 played in promoting malignancy in non-small cell lung cancer (NSCLC) remains unclear. In addition, the correlation of EZH2 overexpression and the prognosis of NSCLC patients in non-Asian cohort need to be determined.

**Methodology/Principal Findings:**

Up-regulation of EZH2 was found in NSCLC cells compared with normal human bronchial epithelial cells by western blot assay. Upon EZH2 knockdown using small interfering RNA (siRNA), the proliferation, anchorage-independent growth and invasion of NSCLC cells were remarkably suppressed with profound induction of G1 arrest. Furthermore, the expression of cyclin D1 was notably reduced whereas p15^INK4B^, p21^Waf1/Cip1^ and p27^Kip1^ were increased in NSCLC cells after EZH2-siRNA delivery. To determine whether EZH2 expression contributes to disease progression in patients with NSCLC, Taqman quantitative real-time RT-PCR was used to measure the expression of EZH2 in paired tumor and normal samples. Univariate analysis revealed that patients with NSCLC whose tumors had a higher EZH2 expression had significantly inferior overall, disease-specific, and disease-free survivals compared to those whose tumors expressed lower EZH2 (P = 0.005, P = 0.001 and P = 0.003, respectively). In multivariate analysis, EZH2 expression was an independent predictor of disease-free survival (hazard ratio  = 0.450, 95% CI: 0.270 to 0.750, P = 0.002).

**Conclusions/Significance:**

Our results demonstrate that EZH2 overexpression is critical for NSCLC progression. EZH2 mRNA levels may serve as a prognostic predictor for patients with NSCLC.

## Introduction

Lung cancer is the leading cause of cancer-related deaths in the United States, it kills more that 160,000 Americans each year [1], of which, non-small cell lung cancer (NSCLC) accounts for more than 85% of the cases. Despite ongoing improvements in surgical techniques and chemoradiation therapy, the 5-year survival rate of patients with advanced stages NSCLC was not dramatically improved due to lack of effective treatments [2]. Similar to other cancers, multiple steps that resulted in the accumulation of genetic and epigenetic alternations were involved in the initiation and progression of lung cancer [3], [4]. Therefore, understanding the molecular mechanism of cancer progression is critical for advancing the treatment of lung cancer [5], [6], [7].

Methylation of CpG islands in the promoter regions is a common epigenetic mechanism to inactivate tumor suppressor genes, such as *p16, PTEN, DAPK* [8], [9]. Polycomb group proteins (PcG proteins) are important epigenetic regulators of genes involved in cell proliferation, differentiation, pattern formation and stem cell renewal [10], [11], [12]. EZH2 is a member of PcG proteins and part of the polycomb repressor complex (PRC) 2 which methylates histone H3 at lysine 27 (H3K27Me3). H3K27Me3 will in turn serve as a landmark for recruitment of other protein complexes, such as PRC1, to mediate epigenetic silencing [13]. It was shown that PRCs can recruit DNA methyltransferases directly and induce DNA methylation [14], [15]. Overexpression of EZH2 has been observed in multiple tumor types [16]–[25], and associated with poor prognosis. However, EZH2 appears to have distinct biological roles in different cancer types since it is linked with more favorite prognosis in some cancers [26], [27].

Although EZH2 protein levels was shown to be a negative prognostic indicator in NSCLC [17], [18], the underlying mechanism of EZH2 in promoting malignancy in NSCLC have not been well characterized. In this study, we sought to determine the biological impact of EZH2 ovexpression in NSCLC and to evaluate the possibility of using EZH2 mRNA expression levels as a potential biomarker in predicting clinical outcomes in patients with NSCLC.

## Materials and Methods

### Cell Lines and Patients

NSCLC cell lines (H157, H226, H292, H358, H460, H522, A549, H596, H1299, H1792, H1944, Calu-1, and SK-Mes-1) used in the study were obtained from American Type Culture Collection (Manassas, VA) and were grown in DMEM medium with 10% fetal bovine serum (Mediatech, Manassas, VA ). The normal human bronchial epithelial cell lines HBE1, HBE2 and HBE3 (kindly provided by Dr. John Minna of The University of Texas Southwestern Medical Center, Dallas TX) were cultured in keratinocyte serum-free medium with 25 µg/ml bovine pituitary extract and 0.2ng/ml recombinant epidermal growth factor (Invitrogen, Carlsbad, CA) as described before [28], [29].

Clinical samples included in the study consist of primary tumors and their corresponding nonmalignant lung tissues form 94 individuals with pathologic stage I to IV NSCLC. All of the patients were treated with surgical resection of the primary tumors, except those with stage III and IV tumors who might also receive postoperative radiation therapy and adjuvant chemotherapy, in M.D. Anderson Cancer Center from 1995 to 2000. Samples were immediately frozen and stored at −80°C until analysis. The selection of these patients was based on the availability of archived fresh tumor and corresponding normal lung tissues for the investigators. Clinical information and follow-up information for the study were based on chart review and from reports from the M.D. Anderson tumor registry service. Informed consent for the use of residual resected tissues for research was obtained from all the patients enrolled in the study and all participants provided their written informed consent to be involved in this work. The study was reviewed and approved by the institutional review board of The University of Texas MD Anderson Cancer Center.

### RNA Extraction and Taqman Gene Expression Analysis

Total RNA from tissue samples was extracted by using Trizol reagent according to the manufacturer’s instruction. Approximately 1 to 2 µg of total RNA from each sample was converted to cDNA using SuperScript II reverse transcriptase (Life Technologies, Inc., Gaithersburg, MD) in 20 µL volume. The cDNA product was diluted to 100 µL with sterile water. For Taqman gene expression assay, 2.5 µL of diluted cDNA was mixed with primer limited glyceraldehyde-3-phosphate dehydrogenase (GAPDH) endogenous control probe (Cat # 432631) and EZH2 probe (Assay ID Hs001016789_m1, cat # 4331182) in a total of 25 µL reaction volume and assayed on a 7500 Fast Real-Time PCR system (Applied Biosystems, Carlsbad, CA) according to manufacture recommended condition. The GAPDH-normalized expression level of EZH2 in tumor tissues or adjacent normal lung tissues was calculated as ΔCt (dCt_Tumor or dCt_Normal) = Ct _EZH2_–Ct _GAPDH_. Duplicated runs were performed and the average ΔCt was used for analysis. Using the expression levels relative to the corresponding normal tissues, individual EZH2 level was determined. The fold difference of EZH2 expression in a tumor tissue compared to the corresponding normal tissue is calculated by 2^−ΔΔCt^ (the comparative Ct method), where ΔΔCt = ΔCt Tumor (dCt_Tumor)−ΔCt Normal (dCt_Normal) (25). A fold difference >1 is considered high EZH2 expression in the tumor tissue.

### SiRNA Transfection

Since EZH2 has two major splicing variants, chemically synthesized siRNAs were designed to target both EZH2 splicing variants and purchased from Ambion Inc. (siRNA ID: si-4916 and si-4917). The sequences for these siRNA are 5′- GCUGACCAUUGGGACAGUATT-3′ (si-4916) and 5′-GUGUAUGAGUUUAGAGUCATT-3′ (si-4917). The FAM labeled Scrambled siRNA was also obtained from Ambion Inc. In vitro transient transfection was done using Lipofectamine 2000 (Invitrogen, Carlsbad, CA) following the manufacturer’s protocol.

### Western Blot Analysis

Proteins were harvested from cultured cells with RIPA buffer plus protease inhibitors (Complete Protease Inhibitor, Roche Bioscience). Twenty micrograms of total protein from each sample were separated in sodium dodecyl sulfate- polyacrylamide gel electrophoresis (SDS-PAGE) using a Bio-Rad Mini-Protean II apparatus. The separated proteins in the gel were transferred to nitrocellulose membrane (Schleicher & Schuell BioScience, Keene, NH). The membranes were then blocked with 1% nonfat milk for 30 min at room temperature, incubated with primary antibodies at 4°C overnight, followed by horseradish peroxidase –conjugated secondary antibodies to detect specific immunoreactivity. The antibody against EZH2 (clone 11) were obtained from BD Transduction Laboratories (San Jose, CA). antibodies against Cyclin D1, Cyclin D3, CDK4, CDK6, p15^INK4B^, p21^Waf1/Cip1^, p27^Kip1^, Cleaved PARP Asp214, Cleaved Caspase-3 Asp175, Caspase-3 and GAPDH were obtained from Cell Signaling Technology, Inc. (Danvers, MA ) or Santa Cruz Biotechnology Inc. (Santa Cruz, CA). SuperSignal West Substrate (Pierce Biotechnology, Rockford, IL) was used as the detection agent.

### Cell Proliferation Assay

Cells were transfected with scrambled, si-4916 or si-4917 siRNAs were seeded in a 96-well plate at a density of 1×10^3^ cells per well in triplicate and assayed for cell viability at 24 h interval using a cell proliferation reagent (WST-1, Roche) according to the manufacturer’s instruction. Briefly, 10 µl of WST-1 reagent was added to each well and the plate was incubated at 37°C for 1 h. The absorbance which is an indicator of cell viability was measured at 450 nm. All experiments were independently repeated at least 3 times.

### Cell Cycle Analysis

Cells transfected with siRNA were harvested 72 hours post transfection, washed with phosphate-buffered saline (PBS) and fixed in 70% cold ethanol overnight. Fixed cells were stained with PI/RNase Staining Buffer (BD Pharmingen™) containing 0.1% Sodium Citrate and 0.1% Triton X-100 at 4°C in the dark for 30 min. Cell cycle data were collected at the University of Maryland, Baltimore Flow Cytometry Core and analyzed with FlowJo software.

### Soft Agar Colony Formation Assay

To evaluate the effect of EZH2 knockdown on anchorage-independent growth, 2×10^4^ siRNA transfected cells were mixed with 1.5 ml of 0.35% agarose in DMEM-10%FBS and seeded on top of solidified 0.5% agarose in 6-well plate in triplicate. After solidification of the top layer at 4°C for 30 min, the gel were covered with 1 ml of culture medium and incubated for 3 weeks with medium change every 3 days. At the end of 3 weeks, colonies >0.1 mm in diameter were counted under a microscopic field at ×20 magnification. The mean colony counts were calculated from 6 randomly selected fields for each treatment condition.

### *In Vitro* Cell Invasion Assay

In vitro invasion assay was carried out in BD BioCoat Matrigel invasion chambers (Falcon 354480; BD Biosciences). After rehydration of the chambers, 1×10^5^ transiently transfected cells in 500 µl DMEM containing 1% FBS were added into each of the upper chambers and 750 µl DMEM containing 10% FBS was placed in the lower chamber. After 20 h incubation, the cells were fixed in 4% formaldehyde and stained with 0.5% crystal violet. Non-invading cells on the upper side of the chamber were removed using cotton swab. Invading cells were photographed and counted in 8 randomly selected fields (magnification, ×20). All experiments were performed in duplicate and repeated 3 times.

### Statistical Analysis

Clinical data were summarized using standard descriptive statistics and frequency tabulation. Kruskal-Wallis test and Wilcoxon rank sum test were performed to assess the difference of continuous variables between/among patients’ clinical-pathologic parameters. Correlations between EZH2 mRNA expression and clinical parameters were assessed using Pearson’s correlation coefficient. Survival curves were estimated using Kaplan-Meier method. Univariate Cox proportional hazard model was applied to assess the effect of covariates on overall survival, disease-specific survival (i.e., those who died of lung cancer-related causes specifically), and disease-free survival (i.e., those who developed recurrence and/or metastasis). Multivariate Cox model was used to assess the effect of EZH2 on time to event outcome, adjusting for other covariates shown statistical significance in the univariate analysis. All statistical tests are two-sided and a P*-*value of 0.05 or lower was considered statistically significant. The *t* test for paired data was used for analysis of the *in vitro* studies. All computations were carried out in SAS (Cary, NC) and S-plus 7.0 for Windows (Insightful Corp.).

## Results

### Phenotypic Effects of EZH2 Down-regulation on NSCLC Cells

**Figure 1:**
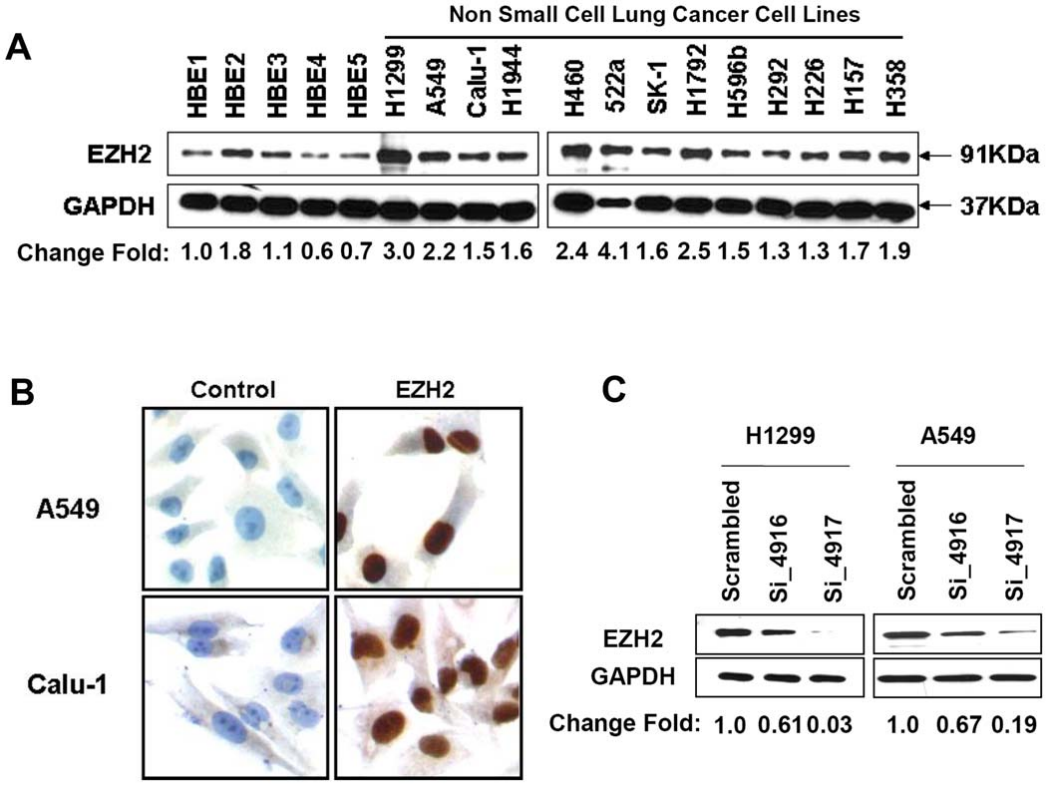
Expression of EZH2 in NSCLC cells and the down-regulation of EZH2 by siRNA. (A) EZH2 expression in NSCLC lung cancer cells and human bronchial epithelia cells (HBEs) were determined by Western blot using whole cell lysate. The blot was first probed with anti-EZH2 antibody and re-probed with anti-GAPDH antibody to indicate the loading quantity. (B) Immunocytochemistry staining of cultured NSCLC cells. A549 and Calu-1 cells were fixed by 1% formaldehyde then stained with anti-EZH2 antibody, followed by detection with anti-mouse secondary antibody and DAB as chromogenic agent. Secondary-antibody only is used as negative control. (C) Example of down-regulation of EZH2 expression by siRNA transfection. H1299 and A549 cells were transfected with EZH2 targeting siRNA si-4916 and si-4917 siRNAs or a scrambled si-RNA control. The whole cell lysate were harvested 72 hours post transfection and analyzed as described in A.

EZH2 protein was detected as a single 91 kDa band on Western blot in all 13 NSCLC cell lines and in immortalized human bronchial epithelia cells (HBEs). The expression in NSCLC cells was generally higher than that in HBEs (Fig. 1A). EZH2 staining was exclusively localized in the nuclei of lung cancer cells (Fig. 1B). H1299 and A549, which had high and moderate levels of EZH2 expression respectively, were selected for additional investigation of the relationship between the EZH2 level and malignant phenotypes.

**Figure 2:**
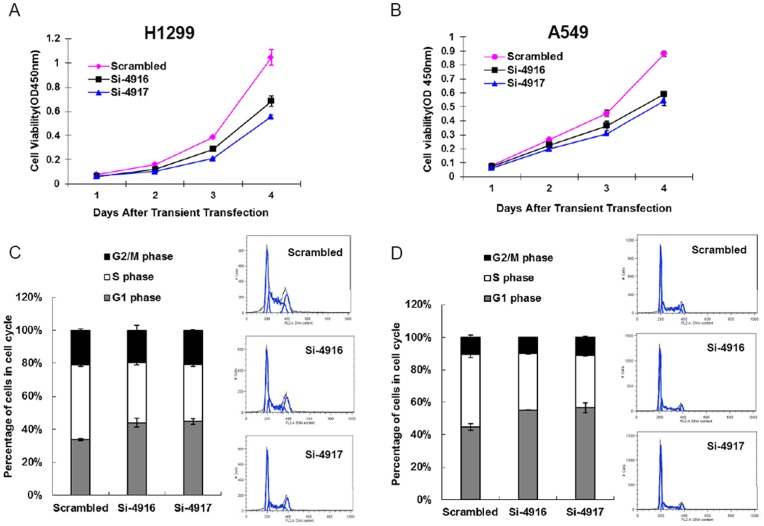
The effect of EZH2 down-regulation on the growth of NSCLC cells. (A, B) Proliferation plot of cells with down-regulated EZH2. A549 or 1299 were transfected with EZH2-targeting siRNA or scrambled siRNA control for 6 hours, then seeded into 96-well plate in triplicate per treatment. The proliferation of the cells was assessed by WST-1 assay at 24 hours interval. (C, D) Distribution of cell population in EZH2 knockdown NSCLC cells. A549 or 1299 cell were transfected with siRNA or scrambled control. The cells were harvested 72 hours later, fixed and stained with propidium iodide. Cellular DNA content were determined by flow cytometry and the distribution of cells is analyzed by FlowJo software.

Two non-overlapping siRNAs (si-4916 and si-4917) were used to silence EZH2 expression in H1299 and A549 cells. Transfection of si-4916 in these NSCLC cells resulted in 10–60% reduction of EZH2 expression, whereas transfection with si-4917 reduced EZH2 protein level by 70–80% (Fig. 1C). Reducing EZH2 expression significantly decreased the proliferation of H1299 and A549 cells (Fig. 2A and 2B). To determine which population of cells was perturbed in cell cycle, flow cytometry analysis was carried out in these transfected lung cancer cells. The results showed that reducing EZH2 expression lead to the accumulation of the cells in G1 phase and the reduction of cells in S phase (Fig. 2C and 2D).

**Figure 3:**
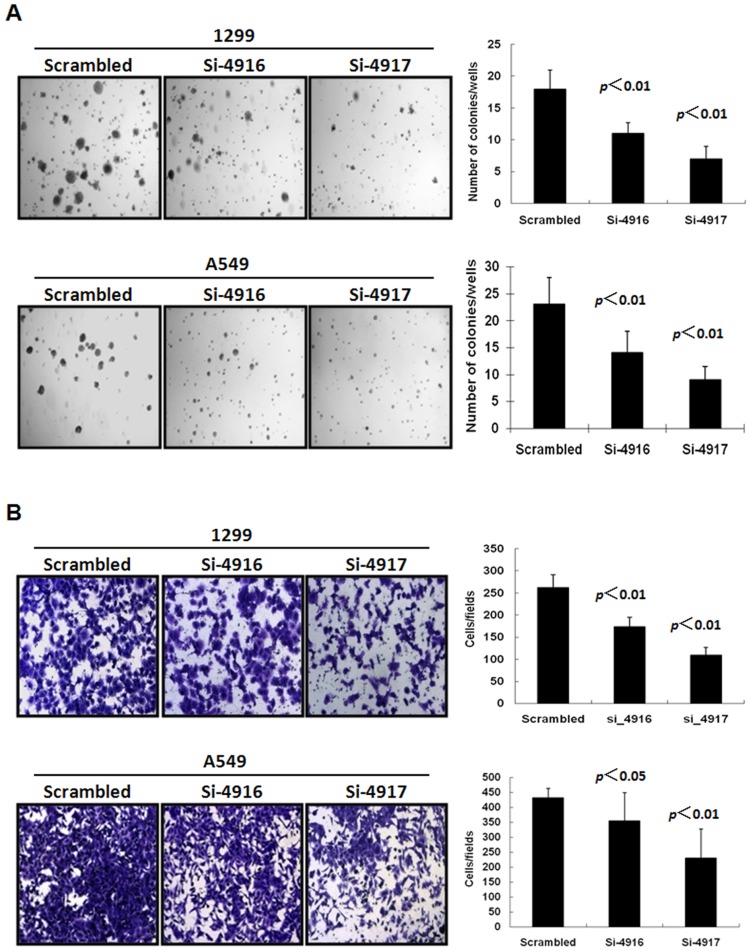
The effect of EZH2 down-regulation on anchorage-independent growth and invasion of NSCLC cells. A549 or 1299 were transfected with EZH2-targeting siRNA or scrambled siRNA control for 6 hours. (A) For anchorage-independent colony formation assay, the transfected cells were seeded in 0.35% agarose in a 6-well plate at 2×10^4^/well. The colonies formed were counted after 3-weeks incubated with regular change of medium. (B) For invasion and migration assay, 1×10^5^ transfected cells in 1% serum were seeded on top of a BD BioCoat Matrigel invasion chamber with 10% FBS in the lower chamber. After 24 hours, the cells were fixed with 4% formaldehyde with 0.5% crystal violet. Cells invaded to the lower chamber were counted under low magnification microscope. The experiments were run in duplicate and repeated three time. The average number and standard derivation of each treatment were graphed here.

In addition, H1299 or A549 cells with down-regulated EZH2 expression showed a significantly reduced colony formation capability. NSCLC cells transfected with si-4916 or si-4917 resulted in 1.5 to 2.8-fold reduction in colonies formed in soft agar as compared to cells transfected with scrambled siRNA control in soft-agar anchorage-independent colony formation assay (Fig. 3A). Furthermore, the ability of these NSCLC cells to invade through extracellular matrix was also significantly reduced upon EZH2 down-regulation as determined by a matrigel coated Boyden chamber invasion assay (Fig. 3B).

### Impact of EZH2 Down-regulation on Cell Cycle Regulators and Metastatic Inhibitor

### Correlation of EZH2 Expression and Clinical Outcome in Patients with NSCLC

To determine whether EZH2 expression contributes to disease progression in patients with NSCLC, we measured EZH2 expression levels in 94 NSCLC tumor tissues and the matching adjacent nonmalignant lung tissues by Taqman quantitative real-time PCR, we then analyzed the correlation of EZH2 expression levels with clinical parameters and treatment outcomes.

Among the 94 patients, 59 patients died and 35 patients were still alive at the time of last follow-up. The median follow-up time is 56-month. Patients’ age at the time of diagnosis ranged from 41 to 84 years, with a median age of 63 years. Forty-one (43%) of the patients were women and fifty-three (56%) were men. Sixty-three (67%) of the patients were current or formal smokers. The histological types of adenocarcinoma, squamous carcinoma, and others were found in 51%, 40%, and 9% of the patients, respectively. Stage I and II patients account for 66% of the all patients, and the remaining 34% patients had Stage III and IV disease. The general clinical and pathological characteristics of the patients are summarized in Table 1.

**Table 1:** Demographic Characteristics of the Patient Population by EZH2 Expression.

Characteristic	N (%)	EZH2-dCt_Normal		EZH2-dCt_Tumor		EZH2-fold change	
		median (range)	*P*	median (range)	*P*	median (range)	*P*
**Gender**							
Male	53 (56.4)	3.7 (7.0, 2.2)	0.230^a^	3.7 (6.6, 1.6)	0.511^a^	0.95 (0.19, 21)	0.198^a^
Female	41 (43.6)	3.9 (6.0, 2.7)		3.7 (8.1, 0.7)		1.07 (0.09, 22)	
Combined	94 (100)	3.9 (7.0, 2.2)		3.7 (0.7, 8.1)		1.00 (0.09, 22)	
**Smoking**							
No	31 (33.0)	3.9 (6.6, 2.2)	0.639	4.4 (8.1, 0.7)	0.100	0.87 (0.22, 22)	0.153
Yes	63 (67.0)	3.9 (7.0, 2.2)		3.6 (7.3, 1.6)		1.18 (0.09, 21)	
**Clinical stage**							
Early(I,II)	62 (66.0)	4.0 (6.6, 2.2)	0.450	3.6 (8.1, 0.7)	0.115	1.13 (0.19, 21)	0.077
Advanced(III,IV,V)	32 (34.0)	3.6 (7.0, 2.2)		4.3 (6.6, 2.1)		0.91 (0.12, 5.8)	
**Pathology**							
Adenocarcinoma	48 (51.1)	4.1 (6.6, 2.2)	0.310^b^	3.9 (6.6, 0.7)	0.458^b^	1.18 (0.18, 22)	0.109^b^
SCC	38 (40.4)	3.6 (2.3, 7.0)		3.9 (8.1, 1.8)		0.95 (0.09, 3.5)	
Large Cell	6 (6.4)	4.0 (5.8, 2.2)		3.7 (7.3, 3.0)		0.50 (0.42, 1.8)	
Other	2 (2.1)	5.1 (5.8, 4.4)		3.5 (3.5, 3.5)		3.01 (1.53, 4.5)	
**Metastasis**							
No met	59	3.9 (6.8, 2.3)	0.724^c^	4.1 (8.1, 1.6)	0.470^c^	0.95 (0.22, 22)	0.328^c^
Intra-Lung	18	3.9 (7.0, 2.2)		3.6 (6.6, 0.7)		0.95 (0.42, 22)	
Brain	7	3.7 (5.2, 3.0)		3.0 (5.3, 1.8)		0.8 (0.52, 1.5)	
Bone	5	3.3 (4.4, 2.9)		3.5 (6.5, 2.2)		1.82 (0.09, 2.9)	
Bone, Liver	1	4.6 (4.6)		3.9 (3.9)		1.65 (1.65)	
Liver, Adrenal	1	4.1 (4.1)		3.7 (3.8)		1.18 (1.18)	
Adrenal	1	3.6 (3.6)		6.6 (6.6)		0.12 (0.12)	
Adrenal, Other	1	5.6 (5.6)		3.3 (3.26)		3.82 (3.82)	
Other	1	5.2 (5.2)		2.7 (2.74)		5.76 (5.76)	

GAPDH normalized EZH2 expression in tumor or adjacent normal lung tissue were represented by dCt_Tumor and dCt_Normal respectively. Higher resulted in smaller dCt value. EZH2-fold change represent expression change in tumor relative to normal lung tissue.

a These p value were from male vs female.

b These p value were from adenocarcinoma vs the others combined.

c These p values were from no metastasis vs all the other with metastasis.

We did not observe significant association between EZH2 expression in tumor or adjacent normal lung and clinicopathological parameters, beside a statistically insignificant association of smoking and EZH2 expression in the tumor (P = 0.1), and a borderline correlation between clinical stage and EZH2 expression in tumor compared to adjacent normal lung tissues (P = 0.07, Table 1). Overall, we found, rather unexpected, that the median level of EZH2 expression in tumor tissue were similar to that of the adjacent non-malignant lung tissues (dCt = 3.86 vs 3.90; median fold change  = 1.0, Table 1).

We then analyzed the associations between EZH2 expression and clinical outcomes of the patients. In the univariate analysis, we observed that the high EZH2 expression in tumor tissue relative to matched adjacent normal lung tissue (high EZH2-fold change) was strongly associated with overall survival, disease-free survival, and disease-specific survival of patients with NSCLC (hazard ratio  = 0.477, 0.414, 0.468 and P = 0.006, 0.002, 0.004 respectively, Table 2). Clinical stage had a marginal association with the disease-free survival (P = 0.090). Kaplan-Meier analysis showed that patients whose tumors had high tumor EZH2 expression relative to paired adjacent normal lung tissue had a significantly inferior clinical outcome compared to those whose tumors had low EZH2 expression. At 5-year post surgery, the probability of overall survival, disease-specific survival and disease-free survival were 25%, 27% and 15%, respectively for the high expression group, comparing to 57%, 59% and 47% for the low expression group (P = 0.005, P = 0.001 and P = 0.003 respectively, Log rank test; Fig. 5A). Interestingly, EZH2 expression level in tumor tissue (dCt_Tumor) by itself is insufficient in predicting the prognosis of patient with NSCLC (Fig. 5B). In multivariate Cox proportional analyses with disease stage as the co-factor, EZH2 expression in tumor tissue relative to matched adjacent normal lung tissue was the independent predictors of disease-free survival (hazard ratio  = 0.045, 95% CI: 0.270 to 0.750, P = 0.002) (Table 2).

**Table 2:** Univariate and multivariate Cox proportional Hazard Model analysis for clinical and pathological factors that may affect survival after resection of NSCLC.

Covariate	Hazard Ratio	95% CI	P value
**UNIVARIATE ANALYSIS**			
**Overall Survival**			
Gender (Female vs Male)	1.487	0.892–2.480	0.128
Smoking (Yes vs No)	1.368	0.770–2.430	0.285
stage (advanced vs early)	1.211	0.709–2.068	0.482
age	1.002	0.975–1.029	0.882
Relative EZH2 expression (high vs low)	0.477	0.282–0.807	0.006
**Disease-Specific Survival**			
Gender (Female vs Male)	1.445	0.847–2.466	0.177
Smoking (Yes vs No)	1.212	0.675–2.174	0.520
stage (advanced vs early)	1.365	0.788–2.362	0.267
age	1.001	0.974–1.030	0.928
Relative EZH2 expression (high vs low)	0.414	0.236–0.725	0.002
**Disease-Free Survival**			
Gender (Female vs Male)	1.185	0.721–1.947	0.504
Smoking (Yes vs No)	1.086	0.639–1.847	0.761
stage (advanced vs early)	1.550	0.935–2.573	0.090
age	0.985	0.959–1.010	0.237
Relative EZH2 expression (high vs low)	0.468	0.281–0.780	0.004
**MULTIVARIATE ANALYSIS**			
**Disease-Free Survival**			
stage (advanced vs early)	1.681	1.011–2.797	0.045
Relative EZH2 expression (high vs low)	0.045	0.268–0.745	0.002

## Discussion

Polycomb group proteins are important for maintaining the spatial patterns of homeotic gene expression that were established early in embryonic development by keeping the silenced state of homeotic genes [30], [31]. Abnormal expression of polycomb group proteins may reshape cellular gene expression pattern. Overexpression of EZH2 has been shown to cause epithelial hyperplasia in mammary glands [32], and to promote malignant transformation of precancerous lesions to cancers in various epithelial tissues [33]–[36]. In addition, increasing evidences suggest that overexpression of EZH2 in cancers contribute to a more aggressive clinical behavior [16], [25], which is likely mediated in part through silencing the expression of cell cycle inhibitors p15^INK4B^ and p16^INK4A^ [37], [38]. However, the precise role EZH2 played in lung cancer progression remains unclear.

In mammalian cells, CDK4/6-cyclin D and CDK2-cyclin E are the primary G1/S cell cycle checkpoint factors controlling the commitment of cells to transit from the G1 phase and enter into DNA synthesis. While CDK4/6-cyclin D and CDK2-cyclin E complexes promote cell entering S phase through relieving inhibition of pRb on transcription of S-phase promoting genes, p15^INK4B^, p16^INK4A^ and p21^Waf1/Cip1^, p27^Kip1^ are negative regulators of these two complexes respectively [39], [40]. In this study, we showed that EZH2 overexpression is required for the growth of NSCLC through promoting G1-S transition; knocking-down the expression of EZH2 in NSCLC cells induced the cells to accumulate in G1 phase while reducing the cells in the S phase. The phenomena were similar to what observed in other types of transformed upper aerodigestive track epithelia cells [33], [41]. The changes in cell cycle distribution is accompanied by reduced expression of cyclin D1, and increased expression p15^INK4B^, p21^Waf1/Cip1^ and p27^Kip1^ in either or both of the NSCLC cell lines, comparable with findings in other tumor types [33], [42], [43], [44], indicating EZH2 may promote malignancy by similar mechanism in different tumor types. The overall effect of EZH2 down-regulation is decreased activities of CDK4/6-cyclin D and CDK2-cyclin E complexes, thus reducing cell proliferation. This suggests one of the major effects of EZH2 dysregulation in NSCLC is to altered cell cycle control.

In addition, we observed EZH2 down regulation significantly impacted other aggressive behaviors of NSCLC cells, such as the ability to form colonies in an anchorage-independent fashion and invading through extracellular matrix. We observed that the expression of DIAB2IP, a metastasis suppressor and a target of EZH2-mediated epigenetic silencing in prostate cancer [45], [46], was increased significantly upon EZH2 knockdown, suggesting a potential role of DIAB2IP in regulating Ras-signaling in lung epithelia cells, and its inactivation as part of the oncogenic process in the lung.

**Figure 4:**
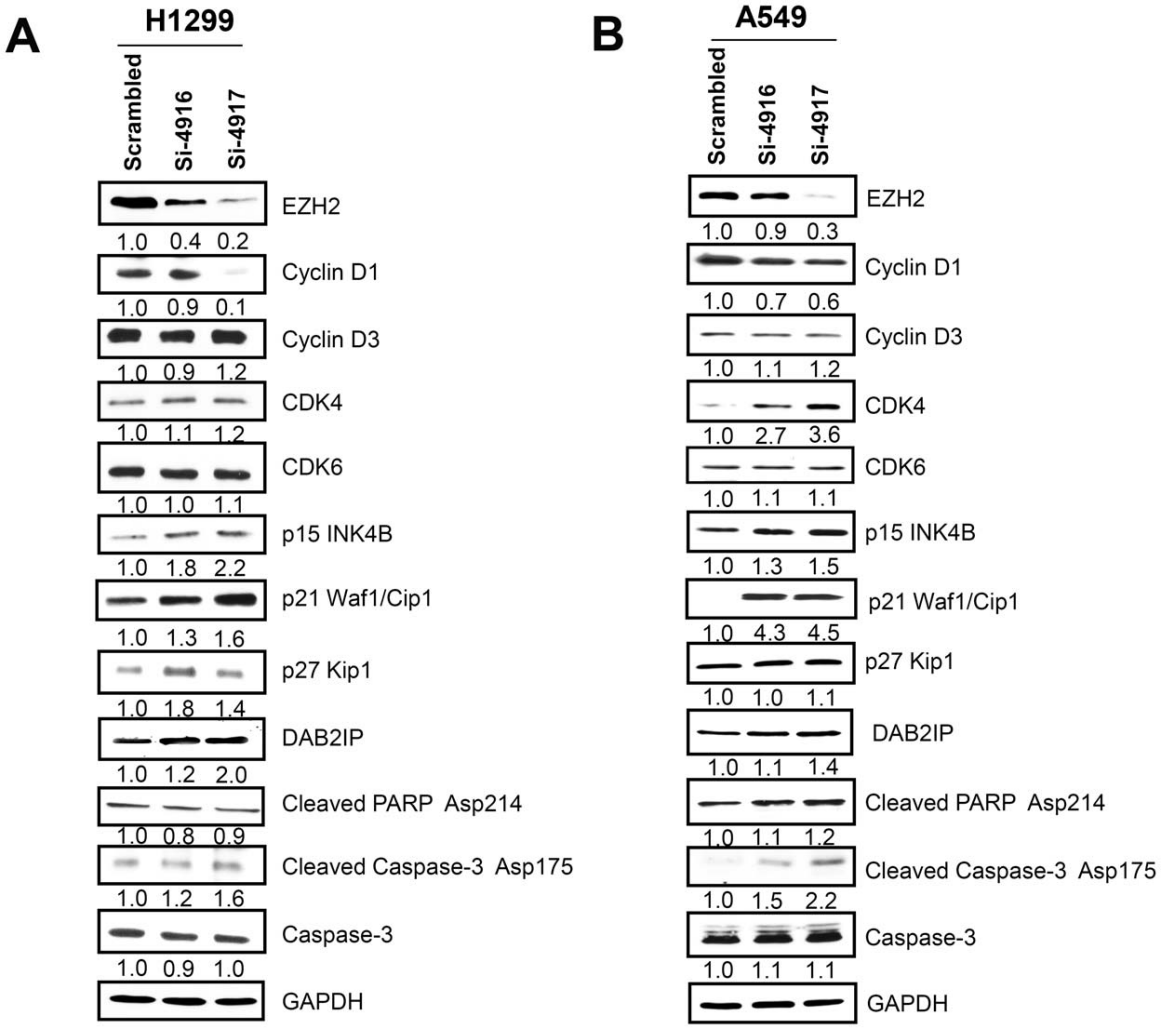
Immnoblot assessment of EZH2 down-regulation on the expression of cell cycle regulators and apoptotic markers. H1299 (A) or A549 (B) cells were transfected with EZH2-targeting siRNA or scrambled siRNA control as indicated. 72 hours post transfection, the cells were harvested and total protein was extracted. Twenty micrograms of protein per well were separated on SDS-PAGE and transferred to nitrocellulose membrane. The blot was probed with the indicated antibody and visualized by HRP-conjugated secondary antibody and ECL. Images on film were scanned using Canon film scanner. The expression level were quantified by digitized pixel density count using Adobe Photoshop and expressed as fold changes beneath the images.

Furthermore, we also observed that expression of caspase 3, apoptotic markers cleaved PARP Asp214 and cleaved caspase-3 Asp175 were increased in A549 cells with EZH2 knockdown (Fig. 4B), suggesting EZH2 overexpression could have anti-apoptosis function in some NSCLC cells.

**Figure 5:**
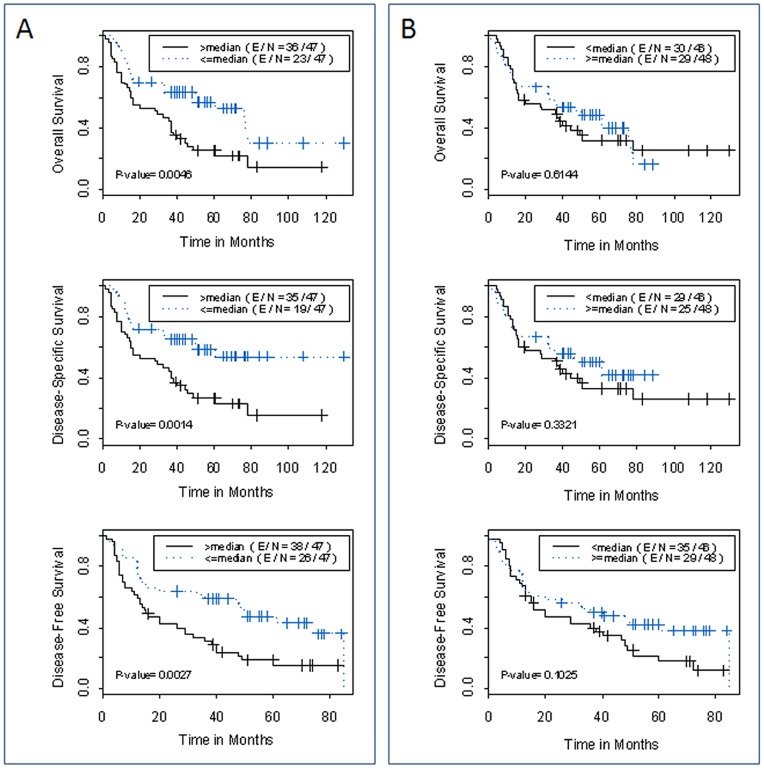
Probability of survival by EZH2 expression level. Kaplan- Meier estimation of overall survival, disease-specific survival and disease-free survival by (A) EZH2 expression in tumor tissues relative to adjacent normal lung tissues (EZH2-fold change); and (B) EZH2 expression levels in tumor tissue by itself (dCt_Tumor). The median of each measure was used to dichotomize the patient population.

Elevated EZH2 immunoreactivity in the tumor was associated with adverse clinical outcome in what appears to be predominantly oriental NSCLC patient cohorts [17], [18]. To determine the correlation of EZH2 expression and the clinical outcome in a US patient cohort, we used quantitative real-time PCR method. Our data indicate that EZH2 expression in tumor relative to adjacent normal lung tissue is a strong and independent predictor of the clinical outcome in these patients. We also observed relatively strong EZH2 expression in tumor adjacent nonmalignant lung tissue, which may result from the chronic carcinogen exposure of the entire lung. Consistent with this possibility, our study showed that EZH2 expression in the tumors of smokers was marginally higher than that in the tumors of none smokers.

A major limitation of our analysis is its relatively small sample size. There for, we were not able to see a strong association of disease outcome with clinical stage, nor did we observe any association of EZH2 expression with the occurrence of metastasis.

In summary, we showed that overexpression of EZH2 contributes to cell cycle deregulation and aggressive phenotypes of NSCLC cells, possibly through altering the cell cycle control mechanism and promoting malignant growth. We also demonstrated that tumor-specific up-regulation of EZH2 mRNA expression is an independent factor for poor survival in patients with NSCLC. These results also suggest that a subset of NSCLC patients whose tumor has a high EZH2 expression may benefit from therapys that target EZH2 [47].
